# The complete mitochondrial genome of *Empoascanara sipra* (Hemiptera:Cicadellidae:Typhlocybinae) with phylogenetic consideration

**DOI:** 10.1080/23802359.2019.1698990

**Published:** 2019-12-13

**Authors:** Chao Tan, Xiaoxiao Chen, Can Li, Yuehua Song

**Affiliations:** a School of Karst Science, Guizhou Normal University/State Engineering Technology Institute for Karst Desertification Control, Guiyang, China;; b Guizhou Provincial Key Laboratory for Rare Animal and Economic Insect of the Mountainous Region, Guiyang University, Guiyang, China

**Keywords:** *Empoascanara sipra*, leafhopper, mitochondrial genome

## Abstract

The complete mitochondrial genome of the leafhopper *Empoascanara sipra* Dworakowska, 1980 was sequenced and annotated from this study. The circular molecule is 14,827 bp in length, including 13 protein-coding genes (PCGs), 22 tRNA genes, two rRNA genes, and one AT-rich region. The base composition of the genome is as follows: A (42.6%), T (33.9%), G (10.0%), and C (13.4%). 11 PCGs have ATN as the start codon, except for *atp8* and *nad5* genes have TTG. The conventional termination codons (TAA or TAG) occur in 11 PCGs, whereas *cox2* and *nad2* use incomplete codon (T––) as termination codon. Phylogenetic analysis using 13 PCGs showed that *E. sipra* was clustered with two Typhlocybinae species, which was consistent with the conventional classification.

Currently, Typhlocybinae comprises almost 5000 described species worldwide, making it the second largest leafhopper subfamily, after deltocephalinae (Dietrich and Dmitriev 2006). The typhlocybine leafhopper species *Empoascanara sipra* belongs to the tribe Erythroneurini with *Empoascanara prima* Distant, 1918 as its type species (Dworakowska 1980; Song and Li 2013). Many leafhopper species in this subfamily are harmful to woody and herbaceous plants through sucking, ovipositing, and virus transmission (Mckamey 2002; Du et al. 2017). In this study, all examined samples were collected from Fanjing Mountain in Guizhou Province of China (N27°52′, E108°47′). The whole body specimen was preserved in ethanol and stored in the insect specimen room of Guizhou Normal University with an accession number GZNU-ELS-2019002.

The circular mitochondrial genome of *E*. *sipra* is 14,827 bp in size. This mitochondrial genome was submitted to GenBank database under accession Number. MN604278. The circular mitogenome contains 13 protein-coding genes (PCGs), 22 transfer RNA genes (tRNAs), two ribosomal RNA genes (*rrnL* and *rrnS*), and one AT-rich region. The gene order and orientation of *E. sipra* were identical with other Hemiptera species (Mao et al. 2017). The base composition values for *E. sipra* mitogenome were 42.6, 13.4, 10.0, and 33.9% for A, C, G, and T, respectively, with an overall A + T content of 76.5%. The AT-skew and GC-skew of this mitogenome were 0.113 and −0.146. Twenty-four genes were oriented on the majority strand (N-strand), whereas the others were transcribed onthe minority strand (J-strand). The *E. sipra* mitogenome has a total of 66 bp intergenic spacer sequences, which is made up of 10 regions in the range from 1 to 26 bp. The largest intergenic spacer sequence of 26 bp is located between *nad5* and *trnH*. Gene overlaps were found at eleven gene junctions and involved a total of 40 bp, the longest 10 bp overlapping located between *trnS_2_* and *nad1*.

Eleven PCGs have ATN as the start codon, except for *atp8* and *nad5* genes have TTG. The conventional termination codons (TAA or TAG) occur in 11 PCGs, whereas *cox2* and *nad2* use incomplete codon (T––) as termination codon. The shortest PCG is *atp8* gene (153 bp) and the longest one is *nad5* gene (1645 bp). The mitogenome harbors the complete set of 22 tRNA genes, ranging from 62 bp (*trnC* and *trnG*) to 71 bp (*trnK*). The *rrnL* gene is located between *trnL_1_* and *trnV*, with length of 1187 bp and A + T content of 81.63%. The *rrnS* gene is located between *trnV* and control region, with a length of 725 bp and A + T content of 80.41%. The control region is located between *rrnS* and *trnI* genes, which is 527 bp in length with an A + T content of 82.92%.

Phylogenetic relationship was constructed on nucleotides sequences of 13 PCGs among *E. sipra* and 13 reference species from four different subfamilies in family Cicadellidae. The result showed that *E. sipra* was clustered with two typhlocybine species (Figure 1), which was consistent with the conventional classification.

**Figure 1.**
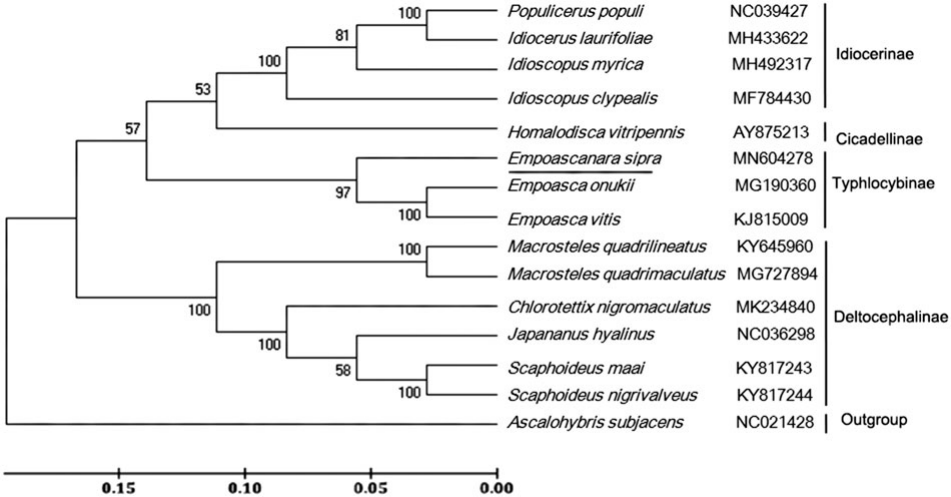
Phylogenetic tree showing the relationship between *E. sipra* and 13 other leafhoppers in inner group based on neighbour-joining method. *Ascalohybris subjacens* was used as an outgroup. GenBank accession numbers of each species were listed in the tree.
